# Powered robotic exoskeletons in post-stroke rehabilitation of gait: a scoping review

**DOI:** 10.1186/s12984-016-0162-5

**Published:** 2016-06-08

**Authors:** Dennis R. Louie, Janice J. Eng

**Affiliations:** Graduate Program in Rehabilitation Sciences, University of British Columbia, Vancouver, BC Canada; Department of Physical Therapy, University of British Columbia, 212-2177 Wesbrook Mall, Vancouver, BC V6T 1Z3 Canada; Rehabilitation Research Program, Vancouver Coastal Health Research Institute, Vancouver, BC Canada

**Keywords:** Stroke, Cerebrovascular accident, Robotic exoskeleton, Gait rehabilitation, Scoping review

## Abstract

Powered robotic exoskeletons are a potential intervention for gait rehabilitation in stroke to enable repetitive walking practice to maximize neural recovery. As this is a relatively new technology for stroke, a scoping review can help guide current research and propose recommendations for advancing the research development. The aim of this scoping review was to map the current literature surrounding the use of robotic exoskeletons for gait rehabilitation in adults post-stroke. Five databases (Pubmed, OVID MEDLINE, CINAHL, Embase, Cochrane Central Register of Clinical Trials) were searched for articles from inception to October 2015. Reference lists of included articles were reviewed to identify additional studies. Articles were included if they utilized a robotic exoskeleton as a gait training intervention for adult stroke survivors and reported walking outcome measures. Of 441 records identified, 11 studies, all published within the last five years, involving 216 participants met the inclusion criteria. The study designs ranged from pre-post clinical studies (*n* = 7) to controlled trials (*n* = 4); five of the studies utilized a robotic exoskeleton device unilaterally, while six used a bilateral design. Participants ranged from sub-acute (<7 weeks) to chronic (>6 months) stroke. Training periods ranged from single-session to 8-week interventions. Main walking outcome measures were gait speed, Timed Up and Go, 6-min Walk Test, and the Functional Ambulation Category. Meaningful improvement with exoskeleton-based gait training was more apparent in sub-acute stroke compared to chronic stroke. Two of the four controlled trials showed no greater improvement in any walking outcomes compared to a control group in chronic stroke. In conclusion, clinical trials demonstrate that powered robotic exoskeletons can be used safely as a gait training intervention for stroke. Preliminary findings suggest that exoskeletal gait training is equivalent to traditional therapy for chronic stroke patients, while sub-acute patients may experience added benefit from exoskeletal gait training. Efforts should be invested in designing rigorous, appropriately powered controlled trials before powered exoskeletons can be translated into a clinical tool for gait rehabilitation post-stroke.

## Background

Stroke is a leading cause of acquired disability in the world, with increasing survival rates as medical care and treatment techniques improve [1]. This equates to an increasing population with stroke-related disability [1, 2], who experience limitations in communication, activities of daily living, and mobility [3]. A majority of this population ranks recovering the ability to walk or improving walking ability among their top rehabilitation goals [4, 5]; furthermore, the ability to walk is a determining factor as to whether an individual is able to return home after their stroke [6]. However, 30 – 40 % of stroke survivors have limited or no walking ability even after rehabilitation [7, 8] and so there is an ongoing need to advance the efficacy of gait rehabilitation for stroke survivors.

Powered robotic exoskeletons are a recently developed technology that allows individuals with lower extremity weakness to walk [9]. These wearable robots strap to the legs and have electrically actuated motors that control joint motion to automate overground walking. Powered exoskeletons were originally designed to be used as an assistive device to allow individuals with complete spinal cord injury to walk [10]. However, because they allow for walking without overhead body weight support or a treadmill, they have gained attention as an alternate intervention for gait rehabilitation in other populations such as stroke where repetitive gait training has been shown to yield improvements in walking function [11, 12]. Several powered exoskeletons are already commercially available, such as the Ekso (Ekso Bionics, USA), Rewalk (Rewalk Robotics, Israel), and Indego (Parker Hannifin, USA) exoskeletons, with more being developed.

There have been many forms of gait retraining proposed for stroke survivors. Conventional physical therapy gait rehabilitation leads to improvements in speed and endurance [13], particularly when conducted early post-stroke [14]. However, conventional gait retraining using hands-on assistance can be taxing on therapists; the number of steps actually taken in a session reflects this and has been shown to be low in sub-acute hospital rehabilitation [15]. Many of the proposed technology-based gait intervention strategies have focused on reducing the physical strain to therapists while increasing the amount of walking repetition that individuals undergo. For example, body weight-supported treadmill training (BWSTT) allows therapists to manually move the hemiparetic limb in a cyclical motion while the patient’s trunk and weight are partially supported by an overhead harness system; this has shown improvements in stroke survivors’ gait speed and endurance compared to conventional gait training [16], yet still places a high physical demand on therapists. Advances in technology have led to treadmill-based robotics, such as the Lokomat (Hocoma, Switzerland), LOPES (University of Twente, Netherlands), and G-EO (Reha-Technology, Switzerland), which have bracing that attaches to the patient’s legs to take them through a walking motion on the treadmill. The appeal of this technology is that it can provide substantially higher repetitions for walking practice than BWSTT without placing strain on therapists; however, there is conflicting evidence regarding the efficacy of treadmill-based robotics for gait training compared to conventional therapy or BWSTT. Some studies have shown that treadmill robotics improve walking independence in stroke [17, 18] but do not improve speed or endurance [18, 19]. There has been some sentiment that such technology has not lived up to the expectations originally predicted based on theory and practice [20]. One argument is that these treadmill robotics with a pre-set belt speed, combined with body weight support, create an environment where the patient has less control over the initiation of each step [21]; another argument against treadmill-based gait training is the lack of variability in visuospatial flow, which is an essential challenge of overground walking [20]. Powered robotic exoskeletons, though similar in structure to treadmill-based robotics, differ in that they require active participation from the user for both swing initiation and foot placement; for example, some exoskeletons have control strategies which will only assist the stepping motion when it detects adequate lateral weight-shifting [9]. Furthermore, because the powered exoskeletons are used for overground walking, it requires the user to be responsible for maintaining trunk and balance control, as well as navigating their path over varying surfaces.

While these powered exoskeletons hold promise, the literature surrounding their use for gait training is only just beginning to gather, with the majority focusing on spinal cord injury [22–24]. Several [25–27] systematic reviews have shown safe usage, positive effects as an assistive device, and exercise benefits for individuals with spinal cord injury. Only one systematic review [28] specifically focusing on powered exoskeletons has included studies involving stroke participants, though studies in spinal cord injury and other conditions were also included. This review focused exclusively on the Hybrid Assistive Limb (HAL) exoskeleton (Cyberdyne, Japan), (which currently is not approved for clinical use outside of Japan), and found beneficial effects on gait function and walking independence; however, the results were combined generally across all included patient populations and not specifically for stroke.

Given that this is a relatively new intervention for stroke, the objective of this scoping review was to map the current literature surrounding the use of powered robotic exoskeletons for gait rehabilitation in post-stroke individuals and to identify gaps in the research. The second objective of this scoping review was to preliminarily explore the efficacy of exoskeleton-based gait rehabilitation in stroke. As this is a relatively new technology for stroke, a scoping review can help guide current research and propose recommendations for advancing the technology.

## Review

### Methods

This scoping review was conducted in accordance with the framework proposed by Arksey and O’Malley [29], and guided by the refined process highlighted by Levac et al. [30].

OVID MEDLINE, Embase, Cochrane Central Register of Controlled Trials, PubMed, and CINAHL databases were accessed and searched from inception on October 14, 2015. We combined the search terms (robot* OR exoskeleton OR “powered gait orthosis” OR PGO OR HAL OR “hybrid assistive limb” OR ReWalk OR Ekso OR Indego) AND (stroke OR CVA OR “cerebrovascular accident” OR “cerebral infarct” OR “cerebral hemorrhage” OR hemiplegia OR hemiparesis OR ABI OR “acquired brain injury”) AND (gait OR walk OR walking OR ambulation), with humans and English language as limits.

Inclusion criteria were full-text, peer-reviewed articles that used a powered robotic exoskeleton with adults post-stroke as an intervention for gait rehabilitation. Articles were included if they reported functional walking outcomes (e.g., speed, distance, independence). We defined a powered robotic exoskeleton as a wearable robotic device which actuates movement of at least one joint while walking, either unilaterally or bilaterally. We further defined powered robotic exoskeletons as stand-alone devices that can be used for overground walking, with programmable control. Articles were excluded if they: reported only technology development; reported only electromyography, physiological cost, or joint kinematic data; combined other interventions (e.g., functional electrical stimulation); included healthy participants or children; utilized a treadmill-based device (i.e., the exoskeleton and treadmill are a single device, where the exoskeleton cannot be used separately overground); included mixed diagnosis participants (<50 % stroke); or if only an abstract was available.

Titles and abstracts were screened for relevance by two reviewers (DRL, CC) according to the inclusion and exclusion criteria above. In the event of conflict, a third reviewer (JJE) was consulted for resolution. Full-texts were then screened and reference lists of all selected articles were searched for additional studies. Included articles were then examined to extract data regarding study design, exoskeleton device, participant characteristics, intervention, training period, outcome measures, adverse effects, and results. We examined the changes in functional walking outcomes relative to clinically meaningful change values published in the literature (Table 1).

**Table 1:** Meaningful change values for functional walking outcomes in stroke

Outcome measure	Sub-acute stroke	Chronic stroke
TUG	Not available	MDC = 2.9 s [44]
6MWT	MDC = 61 m [43]	MCID = 34.4 m [42]
10MWT/gait speed	MCID = 0.16 m/s [45]	MCID = 0.06 m/s (small) [43] MCID = 0.14 m/s (substantial) [43]
FAC	Not available	Not available

*6MWT* six-minute walk test, *10MWT* ten meter walk test, *FAC* functional ambulation category, *MCID* minimal clinically important difference, *MDC* minimal detectable change, *TUG* timed up and go

### Results

**Fig. 1:**
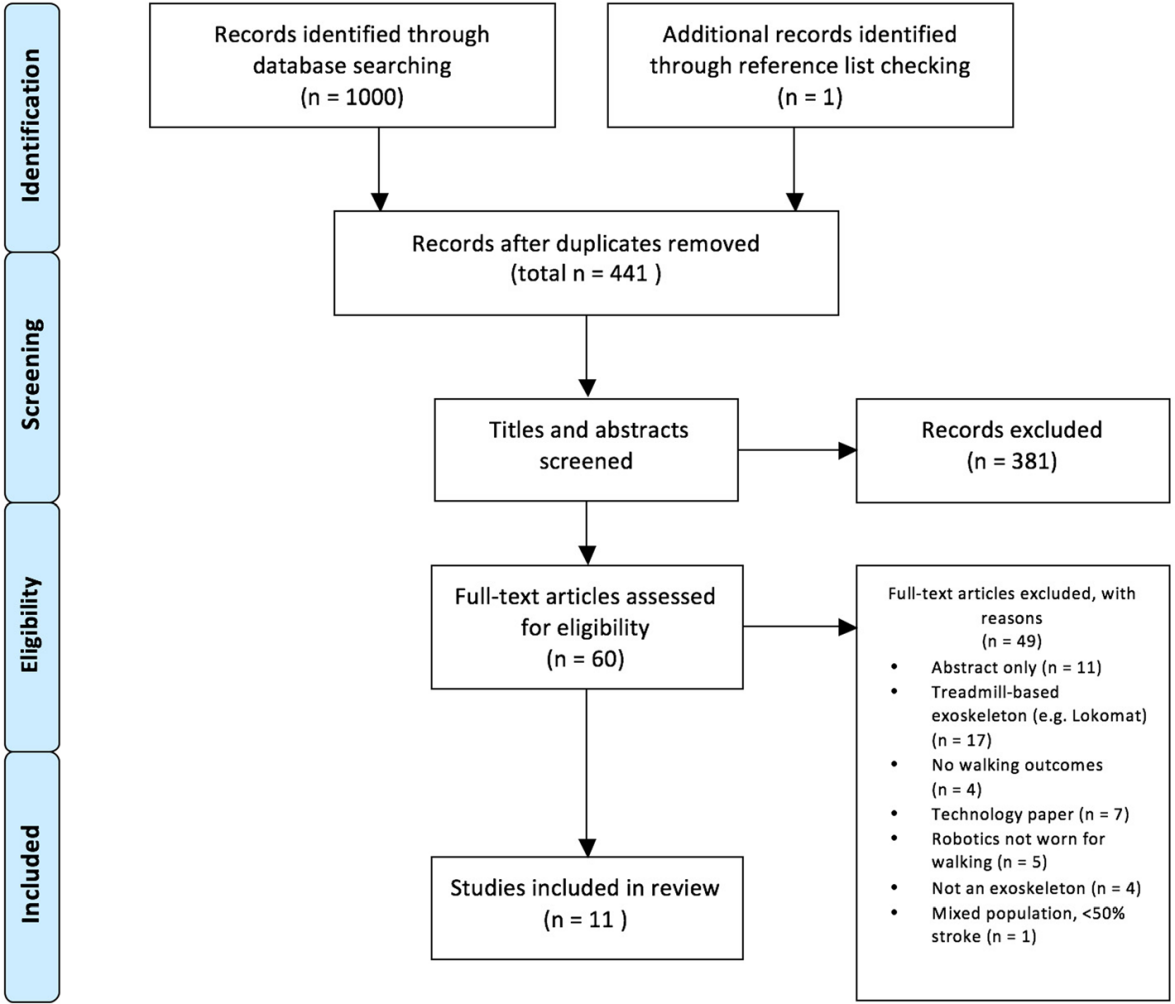
Study results: A flowchart of selection process based on inclusion/exclusion criteria

As seen in Fig. 1, our electronic database search returned 440 unique titles. Only one additional article was identified through reference list searching. After screening titles, abstracts, and full-texts for eligibility, 11 articles were included [31–41]. All 11 articles were published in the last five years, with seven [31–33, 35–37, 39] published in the last two years. Five studies were conducted in the United States, five in Japan, and one in Sweden.

#### Study design

Of the included studies, three were randomized controlled trials (RCTs) [31, 35, 36], and one was a non-randomized controlled study [37]. The rest were a variety of single-group pre-post clinical trials as seen in Table 2. Of the three RCTs, two were smaller in size (*n* = 24 and *n* = 22) and considered pilot studies [31, 36].

**Table 2:** Summary of studies included in the review

Study & Design	Participants	Exoskeleton & Training Period	Training Protocol	Walking outcomes & Results
Subacute Stroke				
Watanabe et al. (2014) [31] Unblinded RCT	Sub-acute stroke 1 – 2 person assist ambulation (HAL group *n* = 11, mean 58.9 days post-stroke Conventional group *n* = 11, mean 50.6 days post-stroke)	HAL – Unilateral 12 sessions over 4 weeks 20 minute sessions	HAL group – gait training while wearing HAL, facilitating improvements in walking ability, partial BWS if needed; progress as able from complete assistance by device to assist-as-needed through bioelectric signal detection Conventional group – facilitate improvements in walking ability, customized to functional level; speed and duration of walking gradually increased	1) TUG – No significant difference in improvement between groups 2) 6MWT – No significant difference in improvement between groups 3) Gait speed – No significant difference in improvement between groups 4) FAC – HAL group improved significantly (p = 0.04) more than Conventional group (change of +1.1 for HAL group; change of +0.6 for Conventional group)
Nilsson et al. (2014) [32] Pre-post study	Sub-acute stroke 1 – 2 person assist ambulation (*n* = 8, 6 – 46 days post-stroke)	HAL – Bilateral 5 sessions/week, median 17 sessions 25 minutes training	Progression from weight shift control to bioelectric signalling control, training with BWS on treadmill; progression of speed and BWS as tolerated	1) 10MWT – median change of +**0.24 m/s**, 4 previously non-ambulatory progressed to ambulatory 2) FAC – median change of +1.5 (from 0 to 1.5)
Fukuda et al. (2015) [33] Pre-post study	Sub-acute stroke (*n* = 53, 12 non-ambulatory, 41 ambulatory)	HAL – Uni/bilateral 2 sessions/week, mean 3.9 sessions	Walking on treadmill in exoskeleton, progress from complete control to bioelectric signalling	1) 10MWT – change of +0.1 m/s for Brunnstrom stage III (greater severity with lower stage) (*n* = 12); no change for Brunnstrom stage IV (*n* = 7); change of +0.1 m/s for Brunnstrom stage V (*n* = 12); change of **+0.4 m/s** for Brunnstrom stage VI (*N* = 10)
Maeshima et al. (2011) [34] Pre-post study	Sub-acute stroke 1 – 2 person assist ambulation (*n* = 16, 27 – 116 days post-stroke)	HAL – Bilateral Single session	Walking and stair practice after standing practice in exoskeleton	1) 10MWT – positive change for 14 of 16 patients (values not provided)
Chronic Stroke				
Buesing et al. (2015) [35] Single-blind RCT	Chronic stroke Limited community ambulation (SMA group – *n* = 25, mean 7.1 years post-stroke Functional task specific training group – *n* = 25, mean 5.4 years post-stroke)	SMA – Bilateral 18 sessions over 6 – 8 weeks 45 minute sessions	SMA group – 30 minutes of high intensity overground walking with SMA (12-16 RPE or 75 % HR max) and 15 minutes of dynamic functional gait training with SMA (varied surfaces, multi-directional stepping, stair climbing, obstacles, community mobility) Functional task specific training group – 15 minutes of high intensity overground walking training and 30 minutes of functional goal-based mobility training	1) Gait speed – No significant difference in improvement between groups
Stein et al. (2014) [36] Single-blind RCT	Chronic stroke Independent ambulation (AlterG group *n* = 12, mean 49.1 months post-stroke Exercise group *n* = 12, mean 88.5 months post-stroke)	AlterG – Unilateral 18 sessions over 6 weeks 60 minute sessions	AlterG group – standardized overground functional tasks including transfers, stepping, turning, reaching, gait training, stairs and curbs while wearing exoskeleton Exercise group – group exercises including relaxation, meditation, self-stretching, active range of motion of upper and lower limbs, minimal gait training (5 min/session)	1) TUG – No significant difference between groups 2) 6MWT – No significant difference in improvements between groups 3) 10MWT – No significant difference in improvement between groups
Yoshimoto et al. (2015) [37] Non-randomized controlled trial	Chronic stroke Independent ambulation (HAL group *n* = 9, mean 92.4 months post-stroke Conventional PT group *n* = 9, mean 80.5 months post-stroke)	HAL – Unilateral 8 sessions over 8 weeks 60 minute sessions	HAL group – 20 minutes of HAL walking per session, with some BWS, walking at speed 1.5-1.7 times max walking speed without device Conventional PT group – exercise to improve walking ability including static and dynamic postural tasks, range of motion, and 20 minutes of overground walking training	1) TUG – HAL group improved significantly compared to Conventional PT group (change of **-11.5 s** for HAL group; change of +0.1 s for Conventional PT group) 2) 10MWT – HAL group improved significantly compared to Conventional PT group (change of **+0.21 m/s** for HAL group; change of -0.02 m/s for Conventional PT group)
Kawamoto et al. (2013) [38] Pre-post study	Chronic stroke (*n* = 16, 1 – 11 years post-stroke, 8 dependent ambulatory, 8 independent ambulatory)	HAL – Bilateral 16 sessions over 8 weeks 20 – 30 minutes training	Overground walking with overhead harness for safety and partial BWS; gradual progression from sit-to-stand to walking (gradually increased intensity by changing speed, duration, BWS, and HAL control mechanism)	1) TUG – mean change of -1.1 s 2) 10MWT – mean change of +0.04 m/s
Bortole et al. (2015) [39] Pre-post study	Chronic stroke Independent ambulation (*n* = 3; 60, 6, 11 months post-stroke)	H2 – Bilateral 12 sessions over 4 weeks 30 minute sessions	Overground walking over a linear track Participants in charge of speed and encouraged to walk as much as possible, with breaks	1) TUG – change of +1.7 s, -2.5 s, -2.5 s 2) 6MWT – change of **-115 m**, +16 m, **+103 m**
Byl et al. (2012) [40] Pre-post study	Chronic stroke Independent ambulation (*n* = 3; 6, 1.3, 10 years post-stroke)	AlterG – Unilateral 2 – 4 sessions/week over 4 weeks 90 minute sessions	Walking practice, with sit-to-stand transfers, squatting, and stepping activities; obstacle clearance, uneven terrain, community ambulation, stair climbing	1) TUG – change of **-6.9 s**, +1.9 s, -0.2 s 2) 6MWT – change of **+37 m**, **+47 m**, +29 m 3) 10MWT – change of **+0.21 m/s**, **+0.14 m/s**, **+0.20 m/s**
Wong et al. (2011) [41] Pre-post study	Chronic stroke Independent ambulation (*n* = 3; 37, 26, 40 months post-stroke)	AlterG – Unilateral 18 sessions over 6 weeks 60 minute sessions	45 minutes while wearing device, standardized weight-bearing functional mobility activities, sit-to-stand transfers, balance exercises, gait practice at various speeds on different surfaces, functional task practice	1) TUG – change of -**11.7 s**, -2.3 s, **-4.2 s** 2) 6MWT – change of +17 m, +14 m, +15 m 3) 10MWT – change of -0.01 m/s, +0.05 m/s, **+0.13 m/s**

*6MWT* six-minute walk test, *10MWT* ten meter walk test, *BWS* body weight support, *FAC* functional ambulation category, *H2* H2 exoskeleton, *HAL* hybrid assistive limb, *HR* heart rate, *SMA* stride management assist system, *PT* physical therapy, *RCT* randomized controlled trial, *RPE* rate of perceived exertion, *TUG* timed up and go

**Bold** indicates value surpasses established meaningful change score detailed in Table 1

#### Participants

Across the 11 studies, there was a total of 216 (male/female:136/80) participants with stroke enrolled (Table 2), with variability in the inclusion criteria for participation. Seven studies [35–41] included participants with chronic stroke (at least six months post-stroke). Four studies [31–35] investigated the exoskeleton with sub-acute participants (less than six months post-stroke) during inpatient rehabilitation. The majority of participants were in the 50 – 70 age range. Six studies [35–37, 39–41] specifically enrolled participants with the ability to walk without physical assistance from a therapist, permitting walking devices such as a cane or walker, while three studies [31, 32, 34] specified a requirement of needing manual physical assistance to walk. The former studies aimed to improve mobility for ambulatory individuals with chronic stroke, whereas the latter sought to restore independent ambulation for sub-acute stroke participants. The other two studies [33, 38] enrolled participants with a mix of functional levels.

#### Exoskeletons

The included studies investigated a variety of exoskeletons, each having different set-ups and control mechanisms. Five studies [31, 36, 37, 40, 41] used a robotic exoskeleton unilaterally on the affected leg, while another five studies [32, 34, 35, 38, 39] used a bilateral set-up for gait training. One study [33] progressed participants, as they were able, from a bilateral design to a unilateral configuration. The most studied exoskeleton was the HAL, used in six studies [31–34, 37, 38]; in these studies, participants’ hip and knee joints were electrically actuated in a walking motion. In one study [39] the H2 exoskeleton (Technaid SL, Spain), assisted the hip, knee, and ankle joints. Four studies [35, 36, 40, 41] utilized an exoskeleton powering only one joint of the lower extremity (either hip or knee, uni- or bilaterally); no studies were found in which only the ankle was actuated during gait. Control of the exoskeletons ranged from remote-control button activation [39] to active movement control of stepping; the devices are able to detect movement intention through monitoring joint angles and limb torque [35, 36, 40, 41], or through bio-electric signalling of muscle activity [31–34, 37, 38]. All exoskeletons except the HAL provided supplementary gait assistance on an as-needed basis, in which the user generates as much of the walking movements as possible and the device provides extra torque or support to ensure step completion. The HAL has two modes, one that provides complete stepping assistance and one that adapts to user force generation. Table 3 further details the exoskeletons, their control strategies, and the level of assistance provided.

**Table 3:** Details of powered exoskeletons in this review

Exoskeleton	Joints actuated	Stepping initiation	Stepping assistance
H2 [39]	Hip, knee, ankle	Initiated by hand buttons on walker Pre-set speed	Assist-as-needed for swing
SMA [35]	Hip	Initiated by movement Internal sensors detect hip joint angle to regulate walking	Assist-as-needed for swing
HAL [31–34, 37, 38]	Hip, knee	Initiated by movement (2 modes) Internal sensors detect lateral weight shift Surface electrodes detect muscle activation via bioelectric signals	Full-assistance for swing Assist-as-needed for swing
AlterG [36, 40, 41]	Knee	Initiated by movement Internal sensors detect movement intention via variable force threshold	Assist-as-needed for stance, free swing

*AlterG* AlterG Bionic Leg, formerly Tibion Bionic Leg; *H2* H2 exoskeleton; *HAL* Hybrid Assistive Limb; *SMA* Stride Management Assist system (Honda R&D Corporation, Japan)

#### Training period

There was variability in the training period of the included studies, ranging from a single session [34] to several weeks [31–33, 39, 40] or months [35–38, 41] of training. Training duration lasted from 20 – 90 min per session, and frequency ranged from two to five sessions per week. Table 2 details the different training periods for each study.

#### Training protocol

The training protocol employed in each study differed, and varied depending on the study design, length of the training period, and exoskeleton used (Table 2). Generally, subjects were progressed as tolerated from weight-bearing functional tasks (sit-to-stand, standing balance, weight shifting) to walking practice while wearing the exoskeleton device. Two studies [32, 33] had participants train on a treadmill, which allowed therapists to adjust the walking speed externally. The most detailed training protocols were described in the controlled trials [31, 35–37], wherein individuals were progressed according to various intensity guidelines such as rate of perceived exertion (RPE) [35] and non-exoskeletal walking speed [37]. For example, Yoshimoto et al. [37] advanced the training speed to 1.5-1.7 times the maximal non-exoskeletal 10MWT walking speed before each session. Several studies [31, 32, 37, 38] allowed some body weight support using an overhead harness to improve walking mechanics.

#### Walking measures

Ten of the 11 studies included a measure of gait speed in their assessment of walking ability, either measuring it directly or via the 10-m Walk Test (10MWT). Five studies [31, 36, 39–41] assessed walking endurance by means of a 6-min Walk Test (6MWT), and seven studies [31, 36–41] assessed the Timed Up and Go (TUG) test, which is a measure of functional mobility as it includes sit-to-stand and turning. Two studies [31, 32] also included level of independence or assistance in their assessment of walking ability, using the Functional Ambulation Category (FAC). Participants were not wearing an exoskeleton device when assessed for the above measures in all studies, but gait aids such as canes and walkers were permitted.

#### Effectiveness of exoskeleton-based gait training

Ten studies reported varying degrees of improved walking ability after exoskeleton training (Table 2). Of the four sub-acute stroke studies, only one [31] was a randomized controlled trial (*n* = 22) which showed that participants using the HAL experienced a significant improvement in FAC scores compared to conventional gait rehabilitation matched for training time, no longer requiring manual assistance to walk after the training period (medium effect size). However, they found no significant difference between the HAL intervention and conventional therapy for walking speed or endurance. One small pre-post sub-acute study [32] (*n* = 8) also found an improvement in the median FAC score of their sub-acute participants from 0 (2-person assist to walk) to 1.5 (1-person assist to walk) after exoskeleton-based gait training. Participants in the two other pre-post studies [33, 34] in sub-acute stroke demonstrated improvements in walking speed with only a few sessions, though not all of their participants demonstrated a change greater than the established minimal clinically important difference (MCID) (Table 1).

Across the seven chronic stroke studies, improvements in walking ability were less apparent. In an RCT with 50 participants [35], there was no significant difference between the clinically meaningful improvements in gait speed made by participants in either the exoskeleton or functional training group matched for training time. Similarly, participants using the AlterG Bionic Leg (AlterG, USA) did not demonstrate significant improvements compared to the control group or to baseline after 18 training sessions in a small RCT with 24 participants [36]. In contrast, a nonrandomized controlled trial [37] found significant and clinically meaningful improvements in gait speed and TUG time after training using a HAL compared to conventional physical therapy; however, the control group did not receive the same number of exercise sessions. One larger pre-post study [38] (*n* = 16) did not find changes in gait speed that were beyond the established MCID (Table 1) while three small pre-post studies [39–41], each with three participants, found varying results. Clinical improvements in endurance were made by four participants in two of the pre-post studies [39, 40], using a minimal clinically important difference of 34.4 m in the 6MWT. [42] Three participants across the three smaller pre-post studies [39–41] made meaningful improvements in TUG scores. Four participants in two of the pre-post studies [40, 41] demonstrated a clinically meaningful improvement in walking speed, using an MCID of 0.06-0.14 m/s [43].

#### Adverse effects

Eight studies confirmed that no adverse events occurred during the course of the gait training intervention. One study [32] reported minor and temporary adverse effects such as skin irritation and pain from cuffs and bioelectric detection electrodes. Two studies [33, 34] did not report on adverse events. No studies reported adverse effects on the therapists.

### Discussion

This scoping review was conducted to map the literature surrounding the use of powered robotic exoskeletons for gait retraining for individuals after stroke and to identify preliminary findings and areas where further research is required. This is a relatively new application of powered exoskeletons, as they have only recently become available for clinical use. As expected, there are only a small number of studies published relevant to this topic.

There were four different powered exoskeletons utilized amongst the included studies, ranging from unilateral, single joint devices to bilateral, multi-joint robotics with the capacity to detect volitional bioelectrical signals to initiate powered movement. Other exoskeletons exist on the commercial market for clinical application that have not yet been investigated for stroke such as the Ekso, Rewalk, and Indego (Parker Hannifin Corporation, USA). Research with these other exoskeletons is required to determine their clinical usefulness and would also strengthen the literature in general support of exoskeleton use for gait rehabilitation in stroke patients. Studies comparing unilateral to bilateral designs may also be another avenue for investigating the efficacy of exoskeletal gait retraining.

The majority of the included studies investigated exoskeleton-based gait training in chronic stroke participants. However, the greatest amount of functional and neurological recovery after stroke occurs in the first six weeks after stroke [3, 7]. In reflection of this, all four studies in the sub-acute phase of stroke reported positive effects of exoskeleton training. Two studies [31, 32] demonstrated improved walking independence with repeated exoskeletal gait training for more limited stroke participants, which is in line with findings using treadmill-based robotics [17]. In another study [33], there was significant improvement in walking speed (0.4 m/s) for stroke participants who had some voluntary motor control, but much less change (0.1 m/s) for those without voluntary control. The magnitude and parameter (ability, speed) of walking improvement may vary depending on the initial functional presentation of the exoskeleton user; furthermore, the spontaneous recovery following stroke is a confounding factor for the improvements reported that has yet to be rigorously controlled for in the current literature.

Study findings were not consistent for chronic stroke participants. All chronic stroke participants included were ambulatory, and so studies investigated changes in gait parameters rather than functional ability. While there were modest, but not consistent changes in the pre-post studies, the more rigorous RCTs [35, 36] did not show a difference from their respective control groups when groups were matched for exercise time and interaction with a physical therapist. Even in studies with longer training protocols [35, 36, 38, 41], there was not a trend for greater improvements. Despite receiving the repetitious practice that is required for motor learning [11, 12], chronic stroke participants do not respond as positively to exoskeletal gait training as sub-acute patients. This is consistent with findings in a systematic review [18] of treadmill-based exoskeleton devices for gait training in chronic, ambulatory individuals with stroke. A possible explanation for this is that once an individual is able to walk, they benefit more from unconstrained walking practice with greater variability and unpredictable challenges [14]. While powered exoskeletons do not require the participant to use a treadmill, they still constrain the user to a stereotyped movement pattern and may thus under-challenge them.

The majority of included studies had small sample sizes, which may have limited the power of their study findings and analysis. In addition to this, the majority of these studies were pilot feasibility or pre-post clinical studies; recruitment and lack of a control group may have introduced bias to their findings. For example, one study [37] used a non-randomized controlled design, where the control group was formed of participants who were less able to attend the study training protocol. These results inform the preliminary evidence in the field and more rigorous, appropriately powered randomized controlled trials will continue to advance the clinical application of powered exoskeletons.

#### Future directions for research and suggestions for clinical practice

From our data synthesis we have identified various considerations when using an exoskeleton for gait retraining and propose several questions for future research:

1. Do non-ambulatory chronic stroke participants experience the same improvement in walking ability as sub-acute stroke participants when using an exoskeleton device for gait retraining?
2. How does initial functional presentation impact the nature of improvement in walking ability when using an exoskeleton device for gait rehabilitation?
3. What is the impact of different exoskeletons (number of joints actuated, level of assistance and control of stepping) on gait rehabilitation in stroke?
4. What is the impact of using a bilateral design compared to a unilateral design for gait rehabilitation in hemiparetic stroke?
5. What is the optimal dose of exoskeletal gait training for stroke patients to regain the most walking ability?
6. How does overground exoskeletal gait training compare to body weight-supported treadmill training?
7. Can exoskeletons be used to safely ambulate 2-person assist patients early after stroke with minimal injury risk to therapists?

Additionally, larger sample sizes and rigorous methodology investigating the efficacy of powered exoskeletons in stroke will further strengthen findings for or against their utilization for gait rehabilitation.

At the moment there is insufficient evidence to advocate in favour or against use of powered exoskeletons in clinical practice. The patient’s acuity and functional presentation need to be considered and the extent of benefit has yet remain to be determined through high quality research. The devices, however, have been shown to be safe and feasible for use with stroke patients. They can be used to mobilize more impaired individuals without physically straining therapists. It thus remains up to therapists to use their own clinical judgement of whether to utilize powered exoskeletons with their patients for gait rehabilitation, considering its application for weight-bearing, standing, and automated walking.

#### Limitations

There are a few limitations with the present review. This review excluded non-English studies, which may have led to an incomplete synthesis of data, given that some exoskeletons are developed in non-English countries such as Japan, Germany, Iran, Israel, and Spain. There was heterogeneity in the studies, especially with variability in the training protocols and exoskeletons utilized (control mechanism, unilateral or bilateral application), which makes interpretation of the results challenging. In addition, type, side, and severity of stroke and comorbid conditions were not considered in this review because of the scarcity of studies in this area. As more research trials in stroke rehabilitation using powered exoskeletons are conducted, a systematic review will be able to address these additional considerations.

## Conclusion

Currently, clinical trials demonstrate that powered robotic exoskeletons can be used safely as a gait training intervention for sub-acute and chronic stroke. Preliminary findings suggest that exoskeletal gait training is equivalent to traditional therapy for chronic stroke patients, while sub-acute patients may experience added benefit from exoskeletal gait training. Efforts should be invested in designing rigorous, appropriately powered controlled trials before it can be translated into a clinical tool for gait rehabilitation post-stroke.

## Abbreviations

10MWT, 10-m walk test; 6MWT, 6-min walk test; BWSTT, body weight-supported treadmill training; FAC, functional ambulation category; HAL, hybrid assistive limb; MCID, minimal clinically important difference; RCT, randomized controlled trial; RPE, rate of perceived exertion; TUG, timed up and go
